# Infection with koala retrovirus subgroup B (KoRV-B), but not KoRV-A, is associated with chlamydial disease in free-ranging koalas (*Phascolarctos cinereus*)

**DOI:** 10.1038/s41598-017-00137-4

**Published:** 2017-03-09

**Authors:** Courtney A. Waugh, Jonathan Hanger, Joanne Loader, Andrew King, Matthew Hobbs, Rebecca Johnson, Peter Timms

**Affiliations:** 1 Faculty of Science, Health, Education and Engineering, University of the Sunshine Coast, 90 Sippy Downs Drive, Sippy Downs, 4558 Queensland Australia; 2 Department of Biology, Norwegian University of Science and Technology, 7491 Trondheim, Norway; 3 Endeavour Veterinary Ecology, 1695 Pumicestone Rd, Toorbul, 4510 Queensland Australia; 4 Australian Museum Research Institute, Australian Museum, 1 William Street, Sydney, NSW 2010 Australia

## Abstract

The virulence of chlamydial infection in wild koalas is highly variable between individuals. Some koalas can be infected (PCR positive) with *Chlamydia* for long periods but remain asymptomatic, whereas others develop clinical disease. *Chlamydia* in the koala has traditionally been studied without regard to coinfection with other pathogens, although koalas are usually subject to infection with koala retrovirus (KoRV). Retroviruses can be immunosuppressive, and there is evidence of an immunosuppressive effect of KoRV *in vitro*. Originally thought to be a single endogenous strain, a new, potentially more virulent exogenous variant (KoRV-B) was recently reported. We hypothesized that KoRV-B might significantly alter chlamydial disease outcomes in koalas, presumably via immunosuppression. By studying sub-groups of *Chlamydia* and KoRV infected koalas in the wild, we found that neither total KoRV load (either viraemia or proviral copies per genome), nor chlamydial infection level or strain type, was significantly associated with chlamydial disease risk. However, PCR positivity with KoRV-B was significantly associated with chlamydial disease in koalas (*p* = 0.02961). This represents an example of a recently evolved virus variant that may be predisposing its host (the koala) to overt clinical disease when co-infected with an otherwise asymptomatic bacterial pathogen (*Chlamydia*).

## Introduction

**Figure 1:**
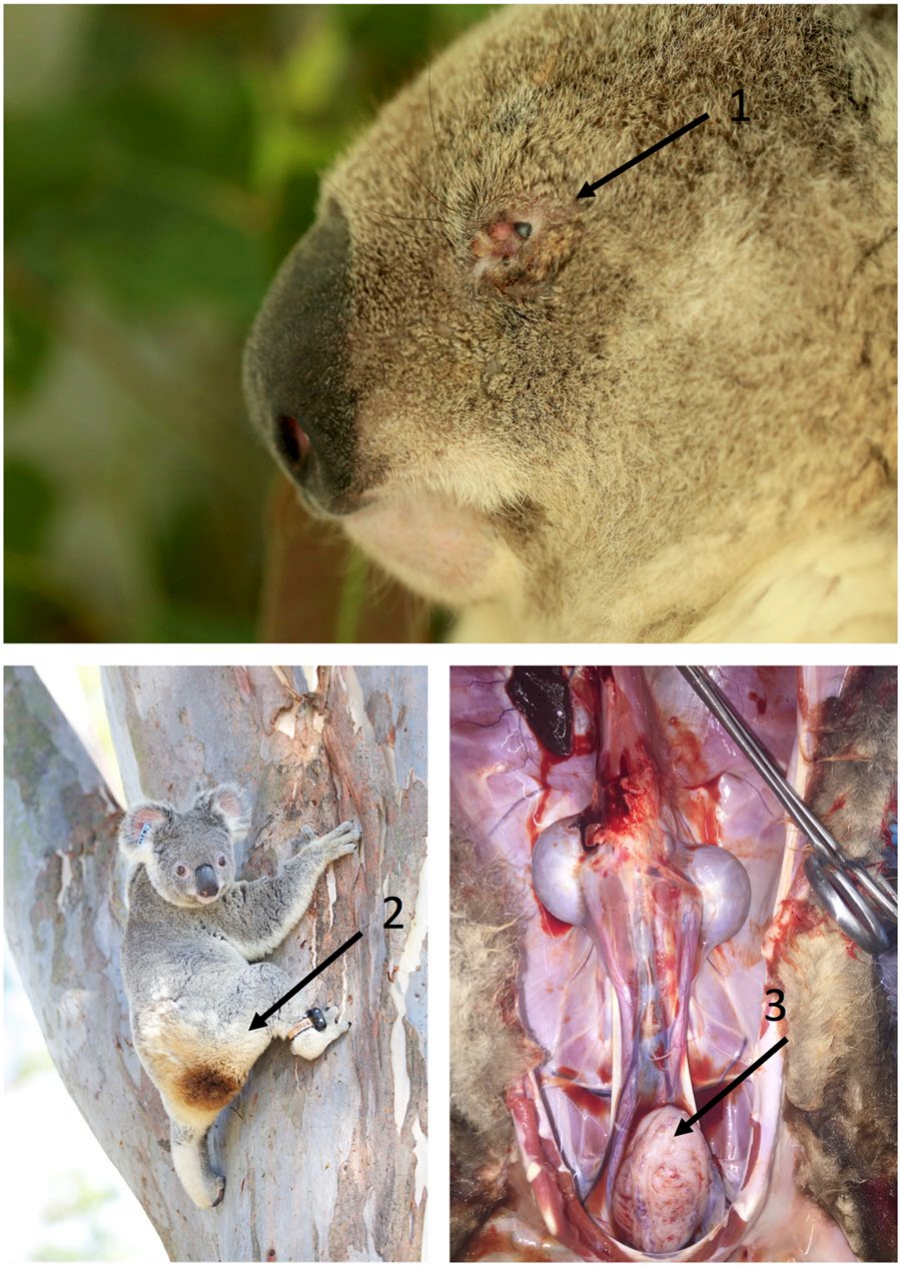
Keratoconjunctivitis (1) and cystitis (bladder inflammation) causing “wet bottom” or “dirty tail” (2) and are two of the clinical manifestations of overt *Chlamydia pecorum* disease seen in koalas (*Phascolarctos cinereus*); and haemorrhage and thickening of the bladder wall (3) is common in chronic cystitis.

*Chlamydia* is an obligate intracellular bacterium with a unique biphasic developmental cycle. Koalas are infected with two species of *Chlamydia*, *C. pecorum* and *C. pneumoniae* ^1^, with *C. pecorum* being the primary pathogen, causing keratoconjunctivitis, reproductive disease, and even death (Fig. 1)^2^. An interesting aspect of koala disease ecology is that the degree of chlamydial virulence varies considerably within and between wild populations. Some *Chlamydia* infected animals present with clinical disease, whereas others remain asymptomatic^3^. What causes this variation in clinical outcomes is unclear. While there is significant diversity in the chlamydial strains that infect koalas^4^, there is no definitive evidence that this strain variation *per se* is the cause of the variation in disease outcomes^5–8^. Therefore, host or environmental factors are likely to be involved.

The koala retrovirus (KoRV) is a recently discovered gammaretrovirus^9^. The first sequenced virus, termed KoRV-A, is considered to be in the process of endogenizing into the genome of wild koalas^10^. Recently other variants, e.g. KoRV-J and KoRV-B (believed to be exogenous) have been discovered^11, 12^. Although the subgroups names are different, both subgroups utilize the same receptor, THTR1, and it has been suggested that KoRV-J should be reclassified as KoRV-B. There are key differences between these KoRV exogenous variants and KoRV-A, such that the variants appear virulent or immunosuppressive in nature; captive koalas infected with KoRV-A only, do not develop leukemia/lymphoma, while koalas co-infected with KoRV-A plus KoRV-B do^12^. Based on relatively limited epidemiological studies, the majority of Australia’s wild koalas are infected with KoRV, with 100% of northern koalas infected and only a few populations on the southern mainland of Australia and close off-shore islands remaining uninfected^10, 13^. Recent unpublished data suggests that this pattern of infection is for endogenised KoRV-A, and that the other variants of KoRV (such as KoRV-B) are present at much lower frequencies.

**Figure 2:**
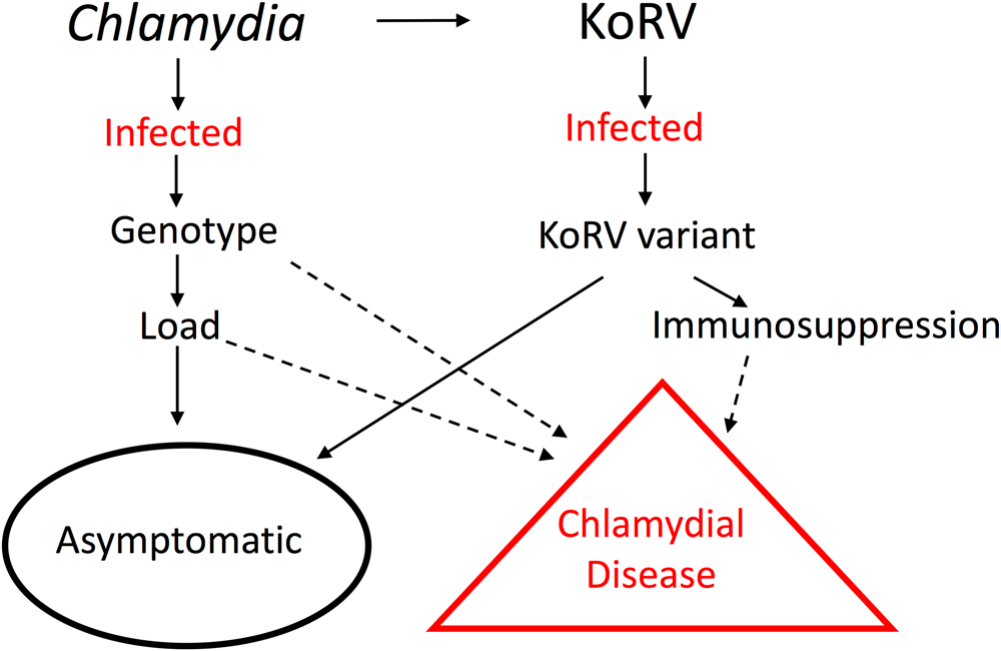
Conceptual model of the role that koala retrovirus (KoRV) may play in the progression of *Chlamydia pecorum* infection to clinical disease in the koala (*Phascolarctos cinereus*). Hypotheses are indicated with dotted arrows.

Many retroviruses, including human immunodeficiency virus (HIV) and feline leukemia virus (FeLV) can induce immunosuppression in their hosts^14, 15^. This increases the host’s susceptibility to opportunistic infections, such as fungal infections (Cryptococcus) and tuberculosis, which are commonly associated with, and exacerbated by retroviruses^16–19^. Evidence for an immunosuppressive property of KoRV has been shown *in vitro* ^20^ but not yet *in vivo*. Previous studies have not been able to demonstrate a statistically significant link between total KoRV viral load and chlamydial disease^21^, but these studies did not seek to differentiate between KoRV variants. As such, we hypothesized that koalas infected with KoRV-B may be immunosuppressed, leading to an exacerbation of chlamydial disease (Fig. 2).

Here we used a model selection approach^22^ to examine the relationship between *Chlamydia* disease outcome and a number of variables, including KoRV load (genomic DNA and viral RNA load) and KoRV variant (KoRV-A and KoRV-B), chlamydial infectious load and genotype, as well as koala age and sex. A key strength of this study comes from our ability to identify tight cohorts of animals for which we have detailed longitudinal data. This allowed us to classify animals into three effective groups for our hypothesis testing: (1) infected with *Chlamydia* but no overt clinical disease; (2) chlamydial diseased; (3) no *Chlamydia* infection and hence no chlamydial disease. We present here the variables that best predict overt signs of clinical chlamydial disease in the koala.

## Results

### Epidemiological characteristics of koala retrovirus (KoRV) in a free-ranging koala population

**Figure 3:**
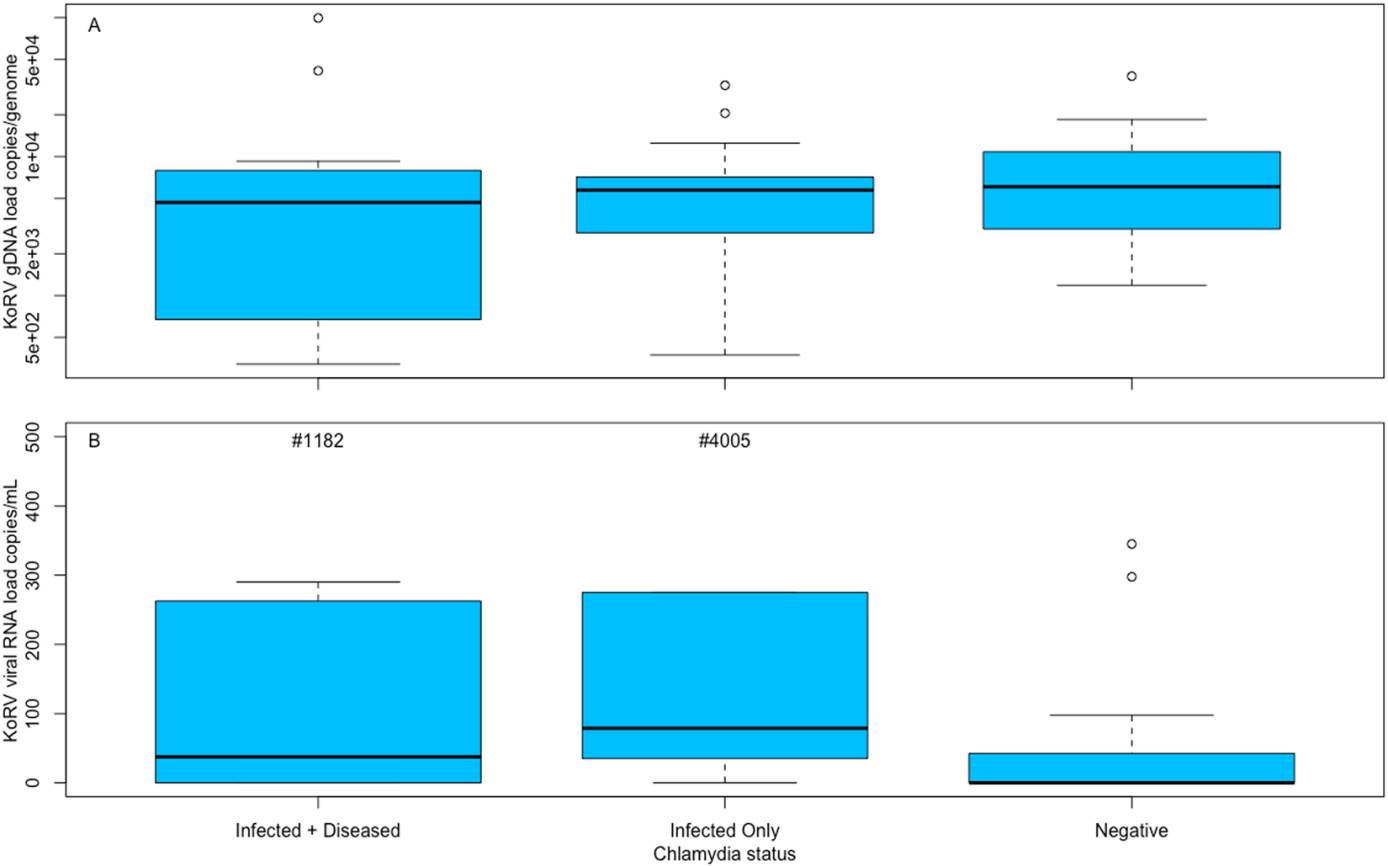
(**A**) Median (Interquartile Range) koala retrovirus (KoRV) genomic DNA load (copies/genome), as measured by qPCR, in DNA extracted from blood in koalas (*Phascolarctos cinereus*) and (**B**) Median (Interquartile Range) KoRV viral RNA load (copies/ul) as measured by qPCR in plasma of koalas. Koalas separated into the following groups: (1) koalas that progress to chlamydial disease (Infected + Diseased; n = 13); (2) koalas that are ‘infected but with no clinical disease’ (Infected Only; n = 10); and (3) Negative animals (that remained *Chlamydia* negative for longer than 12 months; n = 13). The width of the boxes is drawn proportional to the square root of the number of data values. Outliers not shown on axes (but included in analyses) are indicated by #.

Thirty-six free-ranging koalas were analysed as part of our study. Animals included were part of a larger population-wide study by the Queensland Government Department of Transport and Main Roads (as part of the Moreton Bay Rail Link project), conducted between 2013 and 2015 in the Moreton Bay Region, Queensland, Australia. The age of the koalas ranged from 1–9 years, with an average age of 3.4 (+/−0.3) years, with 11 males and 25 females. PCR primer sets designed to amplify part of the variable region A (VRA) and transmembrane p15 domain of the KoRV *env* gene were used to differentiate between KoRV-A and KoRV-B virus types (Table 1). 100% of koalas sampled were positive for endogenous KoRV-A. By comparison, only 25% of the koalas were positive for the variant, KoRV-B (9 of 36 animals). Of the nine KoRV-B positive koalas, all were female with an average (mean +/−SE) age of 3.4 (+/−0.3), ranging between 1–6 years. We also screened all animals (plasma [infectious viral load] and genomic DNA) using KoRV *pol* gene PCR primers, which amplify all KoRV types^21^. Circulating total KoRV viral load in the plasma ranged from 0 to 4005 (242.22+/−115.9) copies/ul while genomic KoRV levels ranged from 3.2 × 10^2^ to 9.9 × 10^4^ copies/genome (1.0 × 10^4^ +/−3.2 × 10^3^; Fig. 3A and B; Supplemental Table S1).

**Table 1:** PCR primers used for koala retrovirus (KoRV) quantification in the blood of free-living koalas (*Phascolarctos cinereus*).

Target	Specificity	Primer Set	Product Size^a^	Reference
env gene	Subgroup A	Universal F: 5′-TCCTGGGAACTGGAAAAGAC-3′ A_R: 5′-GGGTTCCCCAAGTGATCTG-3′	321	This study
env gene	Subgroup B	Universal F: 5′-TCCTGGGAACTGGAAAAGAC-3′ B_R: 5′-GGCGCAGACTGTTGAGATTC-3′	271	This study
pol gene	All KoRVs	F: 5′-TTGGAGGAGGAATACCGATTACAC-3′ R: 5′-GCCAGTCCCATACCTGCCTT-3′	111	Tarlinton *et al.* ^21^

^a^Expected size predicted from alignment with the KoRV subgroup A and B representative genome sequences with Genbank accessions AF151794 and KC779547.

### KoRV-B, but not KoRV-A, is a significant risk factor in the development of chlamydial disease in the koala

In our greater study population of around 500 koalas, for which longitudinal epidemiological data for *C. pecorum* were available, we noted that a *C. pecorum* infection (as detected by PCR) would not always progress to chlamydial disease. We observed that certain koalas, despite having an active (PCR positive) chlamydial infection for 12 months or longer, remained asymptomatic (N = 10; “Infected but no clinical disease” group), whereas other koalas from the same population (N = 13; “Diseased” group) developed overt chlamydial disease (at either the ocular or urogenital site). As a control group, we also selected koalas (N = 13; “Negative” group) that remained *Chlamydia* PCR and *Chlamydia* disease free for the 12-month analysis period; residing within the same population. By utilising these well-defined groups, we were able to model the relationship of the following key explanatory variables: age, sex, KoRV load (in the genomic DNA (gDNA) and viral RNA load in the plasma) and KoRV variant type (KoRV-A and KoRV-B) (Table 2) to chlamydial disease presentation using binary (0 = no infection, or infected but no disease; 1 = diseased) logistic regression (logit link function in the binomial family of general linear models [GLM] (Equation 1).

**Table 2:** Response and explanatory variables for modeling the relationships associated with *Chlamydia pecorum* disease progression in free-living koalas (*Phascolarctos cinereus*).

Variable	Description	Type
DIS	*Chlamydia* disease	Binary response variable 0 = no disease, 1 = disease
AGE	Age of koala	Continuous explanatory variable
SEX	Sex of koala	Nominal explanatory variable
KoRVB	Infected with KoRV B	Two level categorical variable 0 = no, 1 = yes
gDNA	Total provirus load of all KoRVs (copies/cell)	Continuous explanatory variable
cDNA	Total viral RNA load of all KoRVs (copies/μl)	Continuous explanatory variable

**Figure 4:**
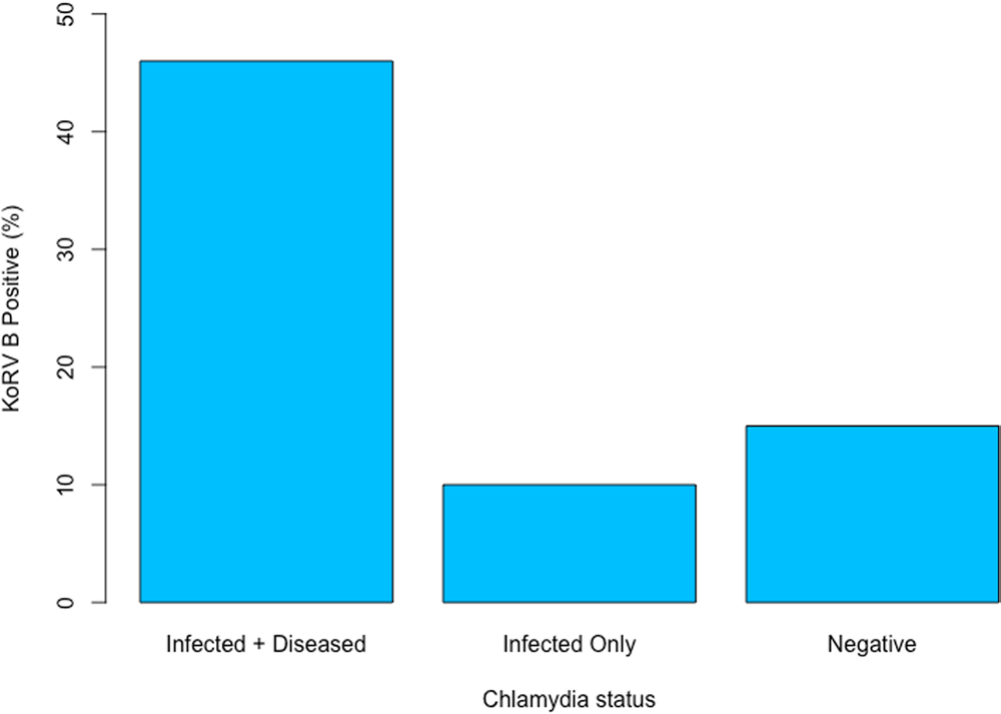
Percentage of koalas (*Phascolarctos cinereus*) infected with koala retrovirus sub type B (KoRV B) versus *Chlamydia pecorum* status. Infected + diseased = animals positive for chlamydial disease (n = 6/13). Infected Only = animals with long term chlamydial infection (as measured by PCR) but that remained asymptomatic/no clinical disease over the one year of the study (n = 1/10). Negative = animals that remained chlamydial negative for longer than 12 months (n = 2/13).

The results of the final model following backwards selection indicated that risk of disease progression in the koala is best explained by presence of the KoRV-B variant in the gDNA. This model was significant, indicating that KoRV-B presence is a significant predictor of chlamydial disease (p = 0.0425; Table 3; Fig. 4). The full results of the original model and the backwards selection are also provided as Supplementary information (S2).

**Table 3:** Estimate and standard error (SE) of the explanation variables in the final model following backwards selection of the binary logistic regression.

Variable	Estimate	SE	Z value	P
Intercept	−0.9985	0.4421	−2.258	0.0239*
KoRV	1.6917	0.8340	2.028	0.0425*

### *C. pecorum* infection load and genotype are not associated with progression to chlamydial disease in the koala

**Figure 5:**
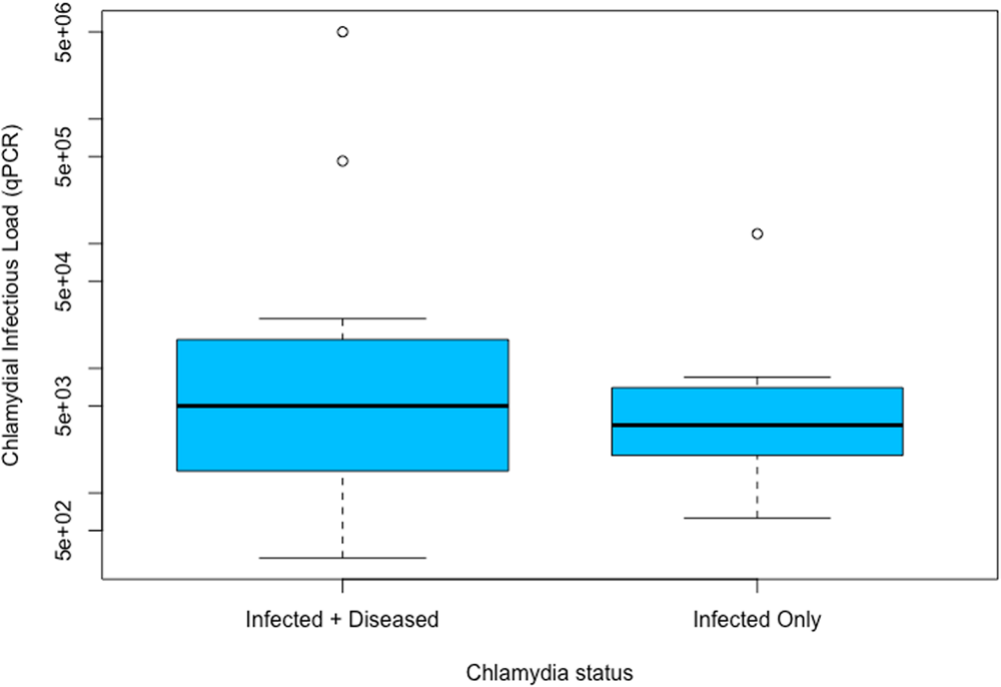
(**A**) Median (Interquartile Range) *Chlamydia pecorum* load (copies/μl) as measured by 16SrRNA qPCR in koalas (*Phascolarctos cinereus*) that progress to chlamydial disease (Infected + Diseased, n = 13) versus koalas that are ‘infected but with no clinical disease’ (Infected Only, n = 10). The width of the boxes is drawn proportional to the square root of the number of data values.

The median (and absolute median deviation [MAD]) chlamydial infection load (as measured by qPCR) in the diseased group (N = 13) was 5571.6 copies/μl. This was slightly higher, but was not found to be statistically significantly different (Wilcoxon-Mann-Whitney test), from the median (MAD) chlamydial load of 2965.2 copies/μl in the “infected but no clinical disease” group (N = 10; W = 73.5, p-value = 0.6188; Fig. 5 and Table 4). Thus, an increased chlamydial load is not seen here to be a significant contributing factor to the development of chlamydial disease pathogenesis in the koala.

**Table 4:** The prevalence (percentage) of *Chlamydia pecorum ompA* genotypes identified in free-ranging koalas (*Phascolarctos cinereus*) inhabiting a population in south-east Queensland, Australia.

Chlamydial *ompA* Genotype^1^	*Chlamydia* infected and diseased % (n)	*Chlamydia* infected but no chlamydial disease % (n)
G	44.4% (4/9)	100% (8/8)
A	11.1% (1/9)	Nil
F	11.1% (1/9)	Nil
E’	33.3% (3/9)	Nil

Koalas are grouped via: *Chlamydia* infected and diseased, n = 9 versus koalas that are infected but with no clinical disease, n = 8).

The *ompA* gene has been used widely as a typing marker for *Chlamydia* ^4, 8, 23–25^ and so we chose this gene to type the *C. pecorum* strains infecting the koalas in this study. Sequence data for *ompA* region VD3-4 were obtained from 17 of the koalas in our study (diseased group: N = 9; infected but no clinical disease group: N = 8). One genotype, G, was commonly present (N = 12 [of 17]; 70.6%) across both groups, and was not related to progression to disease. In the “infected, no disease” group 100% were infected with *ompA* genotype G, indicating that *ompA* genotype G alone is not independently virulent. In the diseased group, 44% of animals were infected with *ompA* genotype G, with 5 of the 9 diseased animals infected with other genotypes; three were infected with *ompA* genotype E’, one animal with *ompA* genotype F, and one animal with *ompA* genotype A (Fig. 5). A two-tailed Fisher’s exact test was then used to quantify how strongly the presence of a particular *ompA* genotype was associated with progression to chlamydial disease. We found that the association between chlamydial *ompA* genotype alone, and disease progression, was non-significant (N = 17; p = 0.0824).

## Discussion

Our data support the current postulate that all northern Australian koalas carry the endogenous KoRV-A strain. In addition, we provide the first data on KoRV-B epidemiology in a free-living northern koala population, with a finding of a 25% prevalence in koalas tested for KoRV-B.

The results of this study further provide new evidence to support a role for KoRV-B in chlamydial disease pathogenesis in the koala. We found that infection with the KoRV-B variant (but not KoRV-A, nor proviral or viraemia levels of total KoRV) was a significant predictor of the development of chlamydial disease in koalas infected with a variety of chlamydial *ompA* genotypes. A previous study investigating the possible link between total KoRV gDNA and RNA load (using the conserved *pol* gene that amplifies all KoRV variants without discrimination) and chlamydial disease also did not detect a statistically significant association^21^. As described above in our model, we similarly found that neither total KoRV gDNA load or KoRV viral RNA load was correlated with chlamydial disease.

Yet, not all koalas that progressed to chlamydial disease were infected with KoRV-B (46%; Fig. 4), and one *Chlamydia*-infected koala that was KoRV-B positive had not progressed to disease at the completion of this study. This indicates that while KoRV-B is a significant component cause, it is neither a necessary nor sufficient cause for chlamydial disease^26^. One explanation for KoRV-B negative koalas that progressed to chlamydial disease could be that they are infected with other exogenous KoRV variants. Exogenous KoRV variants such as KoRV C, D, E, F, have now recently been reported in captive populations of koalas^27, 28^ and could provide further insight into chlamydial disease dynamics.

The non-association of KoRV-A with chlamydial disease may be due to the symbiotic nature of endogenous retroviruses. Endogenous retroviruses, such as KoRV-A, sometimes become symbiotic with their host species and even confer a beneficial or protective effect for their host, as full-length proviruses or partial proviral fragments, through a variety of mechanisms^29^. Some are, however, implicated in recombination events with exogenous variants leading to clinically significant disease and mortality, such as occurs in some FeLV-associated diseases^30, 31^. Hence not all endogenous retroviruses are entirely benign, and recombination events between endogenous KoRV and exogenous variants leading to pathogenicity or greater virulence is possible. Potential mechanisms of virulence of an exogenous KoRV-B, compared to endogenous KoRV-A, may be related to differences in the gp70 surface or p15 transmembrane domains^32^. KoRV-B has duplicated enhancer regions in the LTR, a feature that has been associated with increased virulence in other gammaretroviruses^12, 32^. Further, KoRV-B utilizes a different receptor, the thiamine transporter (THTR1) to infect cells, whereas KoRV-A uses the sodium-dependent phosphate transporter (PIT-1)^11, 12^. These differences, and the possibility of recombination between endogenous and exogenous KoRV variants, provide a number of avenues for further investigation of KoRV effects on immune function in koalas.

Our *Chlamydia* data are consistent with other studies in koalas, as well as in other species, reporting that differences in disease pathogenesis are not always readily explained by *ompA* genotype^8, 23–25^. For example, a large study (n = 820) of southern Australian free-ranging koalas showed that *C. pecorum ompA* genotype was not directly associated with chlamydial disease in koalas^8^. They reported finding both asymptomatic and diseased animals infected with the same dominant genotype (chlamydial *ompA* genotype B). The authors suggested, however, that the dominant presence of chlamydial *ompA* genotype B across Victoria (which is not a dominant genotype in Northern Australia populations) may be less pathogenic than genotypes in the North. This suggestion was based on the observation that there is less disease (lower prevalence and less severe clinical signs) in general reported in the southern versus northern populations of koalas. However, our data suggests an alternative hypothesis for the differences in overt chlamydial disease reported between northern and southern koalas. Due to the significant association found in our study between KoRV-B infection and chlamydial disease progression, we suggest that one explanation for the lower chlamydial disease impacts in the southern koala populations may be due to the lower prevalence of KoRV (probably exogenous variants, such as KoRV-B) in the southern populations^13^.

When assessing the effect of chlamydial genotype on virulence, *ompA* may not be the most appropriate gene to use in order to determine a relationship with disease progression. However, many studies have addressed the diversity of chlamydial strains, using a range of genetic typing approaches, such as *ompA,* MLST, and even whole genome analysis, with no clear associations evident between chlamydial strain diversity and disease outcome^5–7^. The chlamydial plasmid is another factor that has been linked with virulence in some chlamydial species^33, 34^. Yet, two recent studies have specifically addressed this in the koala, but have still not found any significant association with pathology^7, 8^. A limiting factor of these previous studies has been their opportunistic and cross-sectional nature. The current study is the first where we could be confident of cohort characteristics, due to the longitudinal data for each individual koala (>12 months), and provides additional support that chlamydial genotype alone is not strongly associated with disease.

Continuing to understand this co-infection may allow us to provide better treatment options in the future, such as drug and/or vaccine therapies. Currently, adequate treatment regimes for both *Chlamydia* and KoRV do not exist. For *Chlamydia*, antibiotics are often utilized (Chloramphenicol 60 mg/kg/day for up to 45 consecutive days), however, as well as being an impractical solution due to the longevity of treatment, antibiotics affect many of the essential bacteria present in the koala’s gut, which are required for the digestion of eucalypt leaves. KoRV-associated diseases are poorly understood, and there are currently no effective treatments for leukemia, aplastic anaemia and immunodeficiency syndromes in koalas^35^. Vaccines are currently under development for both *C. pecorum* and KoRV in the koala^36–43^ and may prove to be invaluable in the management of declining koala populations, particularly in more easily accessible areas of the eastern seaboard. This discovery of the lower prevalence of potentially pathogenic variants, such as KoRV-B, suggests that while a vaccination schedule against KoRV-A is perhaps not needed (and studies into a beneficial impact of KoRV-A could be useful), a targeted vaccine against exogenous variants, such as KoRV-B, may be an important mitigation strategy to now consider.

## Methods

### Animals

Animals included in the study (n = 36) were part of a larger population-wide study by the Queensland Government Department of Transport and Main Roads (as part of the Moreton Bay Rail Link project), conducted between 2013 and 2015 in the Moreton Bay Region, Queensland, Australia. Koalas in this population have been captured and radio collared in order to be able to undergo veterinary assessments and sampling at 6 monthly intervals^41^, thereby providing a large database of animals for which we had longitudinal datum for each animal. Criteria for inclusion into the study were animals (male and female) of breeding age (>1 year), age was estimated from tooth wear imand growth^44, 45^. Animals were then allocated into the following groups according to their chlamydial disease status: (1) “Infected only”: animals with an active infection for >12 months that remained asymptomatic were designated as “protected” from disease ( N = 10); (2) “Infected + Diseased”: koalas that were infected and progressed to disease within 12 months were designated as “diseased”/non-protected animals; (3) “Negative”: koalas that remained infection and disease free for >12 months were designated as “negative” animals.

### Health assessments and sampling

Ultrasound examination of the kidneys, ureters, urinary bladder and the reproductive tract allowed identification of chlamydial associated urogenital tract diseases including cystitis and reproductive-tract cysts in female koalas. Urinalysis was utilized to detect possible kidney or urinary tract disorders, such as cystitis, which is also associated with *C*. *pecorum*. For the purposes of this study, one set of urogenital (UGT) swabs was collected for *Chlamydia* load and genotype determination, and a blood sample of up to 3 mL was collected from the cephalic vein for KoRV analysis. 500 ul of whole blood was stored at −80 °C for genomic DNA extraction for genomic KoRV determination. Separation of plasma was obtained by centrifugation and 100 ul of plasma was placed into RNALater at −80 °C for viral RNA load determination. All procedures were approved by the University of the Sunshine Coast (USC) Animal Ethics Committee (Animal ethics number AN/A/13/80) and by the Queensland Government (Scientific Purposes Permit, WISP11532912). All experiments were performed in accordance with relevant guidelines and regulations.

### Total KoRV genomic DNA PCR and quantification

Genomic DNA (gDNA) was extracted from whole blood using REDExtract-N-Amp Blood PCR Kit (Sigma Aldrich). Total gDNA (proviral load) load was then quantified using Tarlinton *et al*.^21^ conserved primers (Table 1) using QuantiNova SYBR green kit (Qiagen) as per manufacturer’s conditions. β-actin PCR was performed in parallel with KoRV PCR as a control for DNA quality and to provide relative copy numbers by normalization using this gene. Standards for β-actin and KoRV of known concentration of 10^2^, 10^4^, 10^6^ and 10^8^ copy number of the target gene sequence^12^ were prepared as follows. DNA was extracted from a known KoRV positive koala samples and amplified by PCR. The PCR products were electrophoresed in a 2% agarose/TBE (45 mM Tris-borate and 1 mM EDTA, pH 8.0) gel, stained with ethidium bromide (0.5 ug ml^−1^) and then visualized on a UV transilluminator. The DNA then purified using the High Pure PCR Product Purification Kit (Roche, Applied Science, Germany). Spectrophotometric measurement of absorbance at 260 and 280 nm wavelengths was used to determine the concentration of DNA in the purified preparations. Avogadro’s formula was used to calculate the number of molecules of the product. All reactions were carried out on a Rotor-Gene Q 5-plex HRM platform (Qiagen).

### Total KoRV viral RNA PCR and quantification

Viral RNA was extracted from the plasma using the Qiagen viral RNA mini kit (Qiagen). Contaminating DNA removal and cDNA synthesis of RNA prepared from plasma was conducted using QuantiTect Reverse Transcription kit (Qiagen). Total cDNA (viral RNA load) load was then quantified using Tarlinton *et al*.^21^ conserved primers (Table 1) using QuantiNova SYBR green kit (Qiagen) as per manufacturer’s conditions.

Standards of known concentration of 10^2^, 10^4^, 10^6^ and 10^8^ copy number of the target gene sequence^12^ were prepared as follows. cDNA was prepared, as above, from a known KoRV positive koala samples and amplified by PCR. The PCR product was electrophoresed in a 2% agarose/TBE (45 mM Tris-borate and 1 mM EDTA, pH 8.0) gel, stained with ethidium bromide (0.5 ug ml^−1^) and then visualized on a UV transilluminator. The DNA then purified using the High Pure PCR Product Purification Kit (Roche, Applied Science, Germany). Spectrophotometric measurement of absorbance at 260 and 280 nm wavelengths was used to determine the concentration of DNA in the purified preparations. Avogadro’s formula was used to calculate the number of molecules of the product. All reactions were carried out on a Rotor-Gene Q 5-plex HRM platform (Qiagen).

### KoRV genotyping

**Figure 6:**
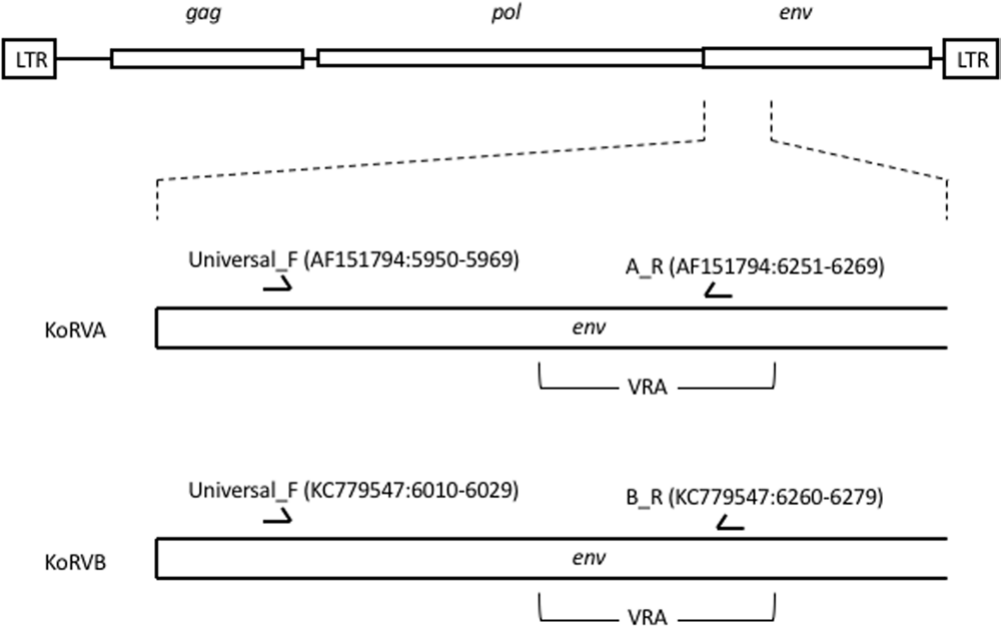
Location of PCR primers used to amplify koala retrovirus KoRV env region from the blood of free-living koalas (*Phascolarctos cinereus*). Primer coordinates are with respect to genome sequences which are representative of KoRV subgroup A (Genbank accession AF151794) and subgroup B (Genbank accession KC779547). Primer sequences are in Table S1. VRA: variable region A; LTR: long terminal repeat.

**Figure 7:**
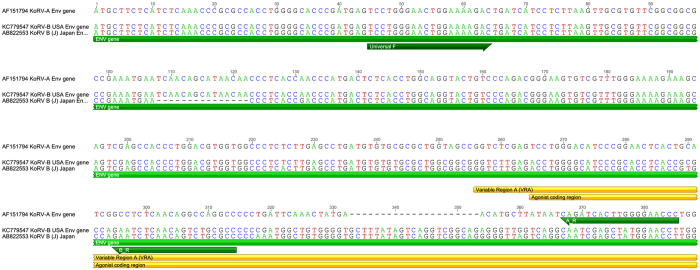
Location of PCR primers used to amplify koala retrovirus (KoRV) env region from the blood of free-living koalas (*Phascolarctos cinereus*). Primer coordinates are with respect to genome sequences which are representative of KoRV subgroup A (Genbank accession AF151794), subgroup B (Genbank accession KC779547), and subgroup J (Genbank accession B22553). Primer sequences are in Table S1. VRA: variable region A; LTR: long terminal repeat.

Primers sets designed to specifically amplify KoRV-A or KoRV-B were used to distinguish between variants (Table 1; Figs 6 and 7). Conventional end-point PCR determination of KoRV-A and KoRV- B from extracted genomic DNA (described above) was determined using HotStartTaq Plus Master Mix Kit (Qiagen) as per manufacturer’s conditions. PCR conditions were as per the following: denaturation of 95 °C for 3 minutes, then 40 cycles of denaturing at 95 °C for 1 min, annealing at 50 °C for 1 min, extension at 72 °C for 1 min, with a final extension at 72 °C.

To confirm the specificity of our KoRV-A specific and KoRV-B specific PCR assays, we sequenced the PCR products from 10 animals positive with the KoRV-A specific PCR and 10 animals positive with the KoRV-B specific PCR (Macrogen Inc.). All PCR products from the KoRV-A amplification matched the published KoRV-A sequence (Genbank accession AF151794). We found equal specificity with our KoRV-B PCR producing sequence that matched the published KoRV-B sequence (Genbank accession KC779547).

### *Chlamydia* quantification

Swab samples were stored at −20 °C until the DNA was extracted as described by Devereaux *et al*.^43^. The extracted samples were screened for the presence of *C. pecorum* using a diagnostic quantitative real-time PCR (qPCR) targeting a 204 bp fragment of the chlamydial 16 S rRNA gene. Assays were as described previously^3, 24^ except for the PCR mixture containing 1 × QuantiTect SYBR Green PCR Master Mix (Qiagen) and 10 μM primers made up to a final volume of 15 μl with PCR-grade water, as well as an increased initial denaturation to 15 mins at 94 °C. All samples were assayed in duplicate. The MC/MarsBar type strain served as a positive control while dH_2_O was used as the negative control. Standards of known concentration of 10^2^, 10^4^, 10^6^ and 10^8^ copy number of the target 16 S rRNA gene sequence were prepared as follows. DNA was extracted from a known positive *C. pecorum* koala samples and amplified by PCR using the 16SrRNA gene real-time primers. The PCR product was electrophoresed in a 2% agarose/TBE (45 mM Tris-borate and 1 mM EDTA, pH 8.0) gel, stained with ethidium bromide (0.5 ug ml^−1^) and then visualized on a UV transilluminator. The band was cut from the gel and the DNA then purified using the High Pure PCR Product Purification Kit (Roche, Applied Science, Germany). Spectrophotometric measurement of absorbance at 260 and 280 nm wavelengths was used to determine the concentration of DNA in the purified preparations. Avogadro’s formula was used to calculate the number of molecules of the product. All reactions were carried out on a Rotor-Gene Q 5-plex HRM platform (Qiagen).

### *Chlamydia* genotyping

Here we employed the molecular target *ompA*, which encodes the *Chlamydia* major outer membrane protein. The nucleotide sequence of *ompA,* which has four variable domains, has been used frequently to genotype *C. pecorum* samples collected from koalas, leading to the detection of 11 koala-associated genotypes, named A-K^4, 23^. To evaluate the level of genetic diversity in the MOMP-encoding *ompA* gene from koala *C. pecorum* strains, *C. pecorum* positive DNA samples detected by our qPCR screening of koala swab samples were used as a template for conventional PCR amplification of the VD3 and VD4 regions of the *ompA* gene (359 bp) for each sample. Primers used in this reaction were CpeNTVD3 (5′-GTTCTTTCTAACGTAGC-3′) and CpeNTVD4 (5′-TGAAGAGAAACAATTTG-3′). PCR conditions were a single cycle of initial denaturation at 95 °C for 5 min, 40 cycles of denaturation at 95 °C for 30 s, primer annealing at 54 °C for 40 s, primer extension at 72 °C for 90 s, followed by a final extension at 72 °C for 7 min. *ompA* sequences were determined by direct sequencing of the PCR product using CpeNTVD3/CpeNTVD4 performed by Macrogen (Korea).

### Statistics

To examine the consequence of KoRV infection status on chlamydial disease progression, we modelled the relationships as a binary logistic regression, using Generalized Linear Models (GLM). We coded the koala’s chlamydial disease status as a binary response variable (0 = diseased, 1 = non-diseased). Diseased koalas were classified as koalas that were infected and had outward signs of clinical chlamydial disease. Non-diseased animals were classified as animals that were not exhibiting clinical signs of *C. pecorum*; this included animals that were either infected with *C. pecorum* with no disease, or had no infection nor disease. >From the information we collected for each incident we prepared explanatory variables such as age, sex, KoRV-B presence, total gDNA KoRV load, and total cDNA KoRV load (Table 2). We followed standard procedures for data exploration^46^ and data presentation^47^. To model the presence of chlamydial disease in koalas as a function of the covariates we fitted a binary logistic regression, using the logit link function in the binomial family of GLM (Equation 1)^48^. We used an information-theoretic approach^49^ to identify a minimum adequate model using backwards selection. Our sample size was relatively small (n = 36) and thus we used Akaike Information Criteria corrected for small samples (AIC). The package glm in the software R (R Core Team 2014) was used to fit the model in Equation (1).

1 $$\begin{matrix} &  & DIS\sim binomial\,({\pi }_{i})\\ E\,(DIS) & = & {\rm{\Delta }}\ge ({\pi }_{i})\\ logit\,(\,{\pi }_{i}) & = & KoRV{B}_{i}+gDN{A}_{i}+\,cDN{A}_{i}+SE{X}_{i}+\,AG{E}_{i}\end{matrix}$$

To examine the impact of chlamydial load on disease pathogenesis, data were first determined to be non-normal via a Shapiro-Wilk test. A Wilcoxon-Mann-Whitney test was then used to determine differences in chlamydial load between the diseased and protected group. A two-tailed Fisher’s exact test was used to quantify how strongly the presence of a particular *ompA* variant was associated with progression to chlamydial disease. All statistics were conducted using R version 3.2.3.

## Electronic supplementary material

Supplementary Information
