# Supplementary material for: Infection with koala retrovirus subgroup B (KoRV-B), but not KoRV-A, is associated with chlamydial disease in free-ranging koalas (Phascolarctos cinereus)
Source: Sci Rep. 2017 Mar 9;7:134. doi: 10.1038/s41598-017-00137-4 (PMC5427818; doi:10.1038/s41598-017-00137-4)
Supplement: Supplementary file 1 — Supplementary Information [file 41598_2017_137_MOESM1_ESM.pdf]

Infection with koala retrovirus subgroup B (KoRV-B), but not KoRV-A, is associated with chlamydial disease in free-ranging koalas (*Phascolarctos cinereus*)

Courtney A. Waugh[1,2], Jonathan Hanger[3], Joanne Loader[3], Andrew King[4], Matthew Hobbs[4], Rebecca Johnson[4], Peter Timms[1*]

[1] Faculty of Science, Health, Education and Engineering, University of the Sunshine Coast, 90 Sippy Downs Drive, Sippy Downs, 4558, Queensland, Australia

[2] Department of Biology, Norwegian University of Science and Technology, 7491 Trondheim, Norway

[3] Endeavour Veterinary Ecology, 1695 Pumicestone Rd, Toorbul, 4510, Queensland, Australia.

[4] Australian Museum Research Institute, Australian Museum, 1 William Street, Sydney, NSW, 2010, Australia

* Corresponding Author: Peter Timms; Faculty of Science, Health, Education and Engineering, University of the Sunshine Coast, 90 Sippy Downs Drive, Sippy Downs, 4558, Queensland, Australia; ptimms@usc.edu.au

Table S1. Raw data showing the gDNA load and viral RNA load in wild koalas (*Phascoarctos cinereus*) with, or without, chlamydial disease.

**Chlamydial disease presence (YES/NO)**	**gDNA (copies/genome)**	**Viral RNA (copies/ul)**
YES	1.48E+03	262.5
YES	3.21E+02	112.5
YES	4.14E+04	0
YES	5.84E+03	0
YES	3.53E+03	0
YES	9.25E+03	290
YES	5.93E+03	290
YES	6.39E+02	0
YES	9.93E+04	0
YES	6.63E+03	0
YES	6.84E+02	1182.5
YES	NA	37.5
YES	6.64E+02	42.5
NO	1.25E+04	40
NO	3.74E+02	1007.5
NO	6.87E+03	115
NO	2.96E+03	0
NO	1.72E+03	42.5
NO	7.12E+03	35
NO	6.16E+03	4005
NO	2.05E+04	275
NO	2.83E+03	120
NO	5.31E+03	0
NO	3.79E+04	97.5
NO	1.08E+04	0
NO	8.11E+03	42.5
NO	1.19E+03	0
NO	2.64E+03	0
NO	3.06E+03	0
NO	NA	40
NO	4.71E+03	0
NO	NA	0
NO	1.85E+04	40
NO	3.01E+03	297.5
NO	7.43E+03	345
NO	0.00E+00	0

Table S2. Results of the backwards selection and estimate and standard error (SE) of the explanation variables in each of the models tested (M1-M5) of the binary logistic regression.

**Model**	**Expression**	**AIC**
M1	DIS ~ KoRV + gDNA + cDNA + SEX + AGE	49.195
M2	DIS ~ KoRV + gDNA + cDNA + AGE	47.237
M3	DIS ~ KoRV + cDNA + AGE	48.197
M4	DIS ~ KoRV + AGE	46.236
M5	DIS ~ KoRV	45.747

M1

	Estimate	Std. Error	Z value	P value
(Intercept)	3.436e-01	1.072e+00	0.321	0.749
KoRV	1.254e+00	1.043e+00	1.203	0.229
gDNA	1.257e-05	2.428e-05	0.518	0.605
cDNA	-3.996e-07	1.648e-06	-0.242	0.808
SEX	-1.968e-01	9.621e-01	-0.205	0.838
AGE	-3.604e-01	2.769e-01	-1.302	0.193

M2

	Estimate	Std. Error	Z value	P value
(Intercept)	2.450e-01	9.609e-01	0.255	0.799
KoRV	1.345e+00	9.475e-01	1.419	0.156
gDNA	1.177e-05	2.369e-05	0.497	0.619
cDNA	-3.963e-07	1.679e-06	-0.236	0.813
AGE	-3.542e-01	2.750e-01	-1.288	0.198

M3

	Estimate	Std. Error	Z value	P value
(Intercept)	-6.710e-02	8.737e-01	-0.077	0.9388
KoRV	1.773e+00	8.742e-01	2.028	0.0426 *
cDNA	-3.129e-07	1.655e-06	-0.189	0.8500
AGE	-2.828e-01	2.541e-01	-1.113	0.2657

M4

	Estimate	Std. Error	Z value	P value
(Intercept)	-0.0826	0.8746	-0.094	0.9248
KoRV	1.7974	0.8678	2.071	0.0383 *
AGE	-0.2887	0.2546	-1.134	0.2568

M5

	Estimate	Std. Error	Z value	P value
(Intercept)	-0.9985	0.4421	-2.258	0.0239 *
KoRV	1.6917	0.8340	2.028	0.0425 *
